# A Triboelectric Nanogenerator Based on TPU/PLA for Basketball Motion Monitoring

**DOI:** 10.1002/open.202400241

**Published:** 2024-11-29

**Authors:** Jun Zhang, Shuai Ma

**Affiliations:** ^1^ Department of Physical Education and Research Lanzhou University of Technology Lanzhou 730050 China; ^2^ School of Physical Education Hanyang University Seoul 04763 Korea

**Keywords:** Triboelectric nanogenerators (TENGs), 3D printing, Basketball sports, Self-powered sensor

## Abstract

Sports monitoring equipment that integrates advanced sensing technology has attracted widespread attention in recent years. Hence, we proposed a TPU/PLA 3D printing film triboelectric nanogenerator (TP‐TENG) to monitor basketball player posture for training effectiveness evaluation. By introducing carbon fibers, TPU/PLA/Carbon film with conductive and positive triboelectric properties was prepared. According to results, the peak power density is 58.38 mW m^−2^ when the load resistance of 30 MΩ is matched. Nevertheless, when TP‐TENG encounters wrinkles and continues to work for a period of time, its output performance will tend to stabilize. And the open‐circuit voltage (*V_OC_*), transfer charge (*Q_SC_*), and short‐circuit current (*I_SC_*) increase to 91 %, 92 %, and 92 %, respectively, almost the same as their original values. Furthermore, the TP‐TENG sensor can recognize human posture in different basketball motion posture, including skipping, squatting, walking, running. This work can help to promote the application of 3D printing TENG in basketball sports monitoring.

## Graphical abstract

A TPU/PLA 3D printing film triboelectric nanogenerator (TP‐TENG) was proposed to monitor basketball player posture for training effectiveness evaluation. The TP‐TENG sensor can recognize human posture in different basketball motion posture, including skipping, squatting, walking, running. This work can help to promote the application of 3D printing TENG in basketball sports monitoring.

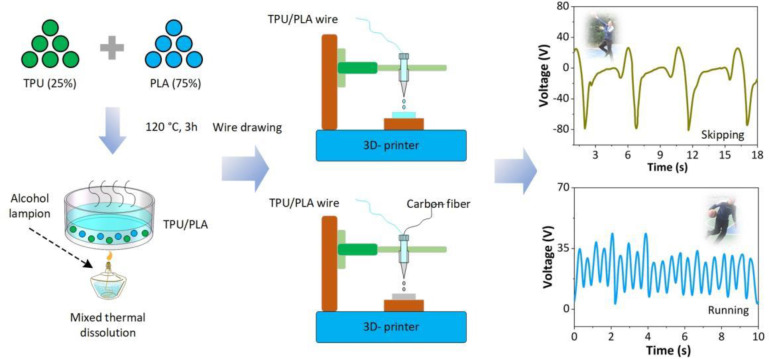

## 1 Introduction

Nowadays, with the development of modern industry and multidisciplinary fields, multifunctional wearable electronic devices used in the field of artificial intelligence technology are receiving worldwide attention.[^1^, ^2^, ^3^, ^4^] The wide application of multi‐functional wearable electronic products has been facilitating our daily life, including in health management, motion detection, drug delivery, electronic skin, and some other aspects.[^5^, ^6^, ^7^, ^8^] In practical applications, wearable devices often face the challenge of surface deformation such as wrinkles due to extreme usage environments.^[9]^ For example, when wearable electronic devices are installed on the human body, they often deform due to various complex postures, such as running, jumping, arm swinging, and other drastic movements.[^10^, ^11^, ^12^, ^13^] Therefore, in practical applications, good flexibility is of great significance for extending service life and improving work stability. Another key issue is that theoretically, wearable electronic products typically require a continuous, sustainable, and easily accessible power system. One potential strategy is to provide a wearable device that is low‐cost, flexible, and light‐weight, and has the conformal energy‐harvesting capability.^[14]^ Triboelectric nanogenerators (TENG) are considered to be one of the most promising power solutions because they are compatible with a variety of materials, have high energy conversion efficiency, and show low dependence on weather and working environment, which can effectively drive wearable electronic devices.[^15^, ^16^, ^17^, ^18^, ^19^, ^20^, ^21^] In recent years, many studies have been focusing on combining TENG technology with wearable electronics, providing more possibilities for continuous, real‐time, and non‐invasive measurement of human physiological and motion signals.[^22^, ^23^, ^24^] However, the simple function of most TENG on the market cannot meet the requirements of multi‐functional wearable electronic products. In addition, in order to obtain a green, stable, and sustainable power supply that is not limited by the working environment, high‐strength performance is crucial for maintaining a beautiful appearance and stable performance.

Moreover, the human skin is a complex sensory system composed of numerous microstructures such as fingerprints, interlocking structures between the epidermis and dermis, as well as various sensory receptors, which can sensitively perceive external static and dynamic pressures, stretching, temperature, humidity, texture, and other stimuli.^[25]^ Recently, the rise of electronic skin has attracted the attention of many researchers due to its ability to achieve sensory functions similar to human skin by converting external stimuli into detectable electrical signals.^[26]^ Among them, the most widely studied is tactile sensors, mainly because they can sense external pressure stimuli and can serve as an important component of simulating human receptors.^[27]^ The advantages of electronic skin have become an important component of electronic skin and have shown great application potential in wearable medical monitoring, intelligent robots, human‐machine interaction, and other fields. Integrating TENG technology with electronic skin is an important way to solve the practical application of TENG.[^28^, ^29^, ^30^, ^31^, ^32^, ^33^, ^34^] Basketball technology refers to various skills and tactics used by basketball players in competitions, including personal skills, team cooperation, tactical application, and psychological qualities.[^35^, ^36^] The improvement of basketball skills plays an important role in athletes’ better performance in competitions. Hence, how to effectively cultivate and improve athletes’ basketball skills has always been a focus of attention for coaches and researchers. However, there are currently some difficulties in basketball technical teaching both domestically and internationally. Nowadays, basketball is developing rapidly, and the pace of basketball matches is getting faster and harder, requiring athletes to have a higher level of basketball skills. The cultivation of basketball skills is not only about technical and tactical training, but also requires the guidance of advanced monitoring techniques, which evaluate the training effect through real‐time monitoring of athletes’ posture. Therefore, it is very meaningful to combine TENG technology with electronic skin and apply it to posture monitoring in basketball training.

In this work, we proposed a thermoplastic urethane/polylactic acid (TPU/PLA) 3D printing film triboelectric nanogenerator (TP‐TENG) to monitor basketball player posture for training effectiveness evaluation. By introducing carbon fibers, TPU/PLA/Carbon film with conductive and positive triboelectric properties was prepared. The TP‐TENG are composed of three layers, including two TPU/PLA/Carbon films and one TPU/PLA film, characterized by lightweight and highly conformal contact. According to results, the peak power density is 58.38 mW m^−2^ when the load resistance of 30 MΩ is matched. Nevertheless, when TP‐TENG encounters wrinkles and continues to work for a period of time, its output performance will tend to stabilize. And the open‐circuit voltage (*V_OC_*), transfer charge (*Q_SC_*), and short‐circuit current (*I_SC_*) increase to 91 %, 92 %, and 92 %, respectively, almost the same as their original values. Furthermore, the TP‐TENG sensor can intelligently test and recognize human posture in different basketball motion posture, including skipping, squatting, walking, running. Clear signal differences help to identify basketball posture and provide a data basis for improving training methods in the later stages.

## Experiments

### Fabrication of TPU/PLA Film and TPU/PLA/Carbon Film

**Figure 1:**
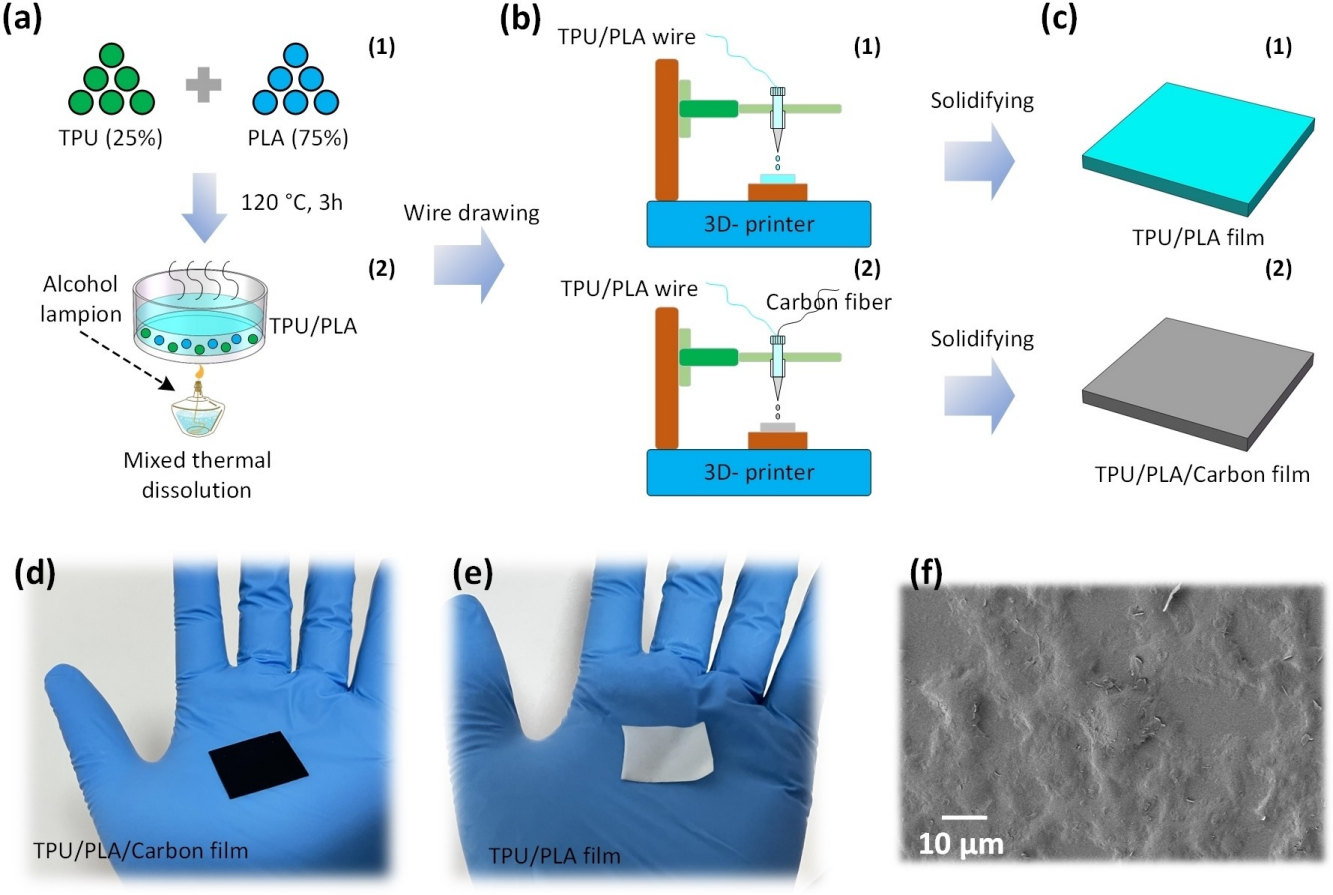
(a1, a2) The fabrication process of 3D printing substrate for TPU/PLA. (b1, c1) The preparation process of TPU/PLA film. (b2, c2) The preparation process of TPU/PLA/Carbon film. The picture of (d) TPU/PLA/Carbon film and (e) TPU/PLA film. (f) The SEM image of TPU/PLA/Carbon film surface.

Figure 1(a1, a2) shows the detailed production process and material configuration of TPU/PLA printing substrate. Specifically, the raw materials and ratio of TPU/PLA are composed of 25 % TPU (Shanghai Oushuo Plastic Co., Ltd, Shanghai) and 75 % PLA (Dongguan Xinshengli Plastic New Materials Co., Ltd, Dongguan). Firstly, dry the two raw material particles continuously for 0.5 hours at approximately 500 °C to eliminate any remaining moisture in the material. Then, the two particles were evenly mixed together and prepared into PLA/TPU filaments with a diameter of about 1.8 mm through a screw stamping mechanism, which were used as the substrate for 3D printing. Figure 1(b1, c1) shows that TPU/PLA is used as a 3D printing substrate for preparing TPU/PLA thin films, and the thickness of TPU/PLA films was 1 mm. Furthermore, we use carbon fiber as the reinforcing phase and PLA/TPU fine wires as the matrix phase to prepare PLA/TPU/Carbon film with both conductivity and triboelectric properties, as illustrated in Figure 1(b2, c2).

### Characterizations and Measurements

Figure 1(d, e) specifically display photos of TPU/PLA/Carbon film and TPU/PLA/Carbon film. Moreover, the surface texture features of TPU/PLA/Carbon film can be observed in the scanning electron microscope (SEM) image, as present in Figure 1(f). The electrical outputs of TP‐TENG, including (*V_oc_*, *I_sc_*, and *Q_sc_*) are measured by using programmable electrometer (Keithley 6514). Also, the long‐term stability test uses a digital oscilloscope (TBS1102X). In detail, TP‐TENG is directly connected to the Keithley 6514, and then Keithley 6514 is connected to the computer terminal through a data acquisition card. The self‐made labVIEW software in the computer can display the V_oc_, I_sc_, and Q_sc_ of TP‐TENG in real time. The data collected by the Keithley 6514 is filtered and processed using LabVIEW software to eliminate the influence of noise.

### Ethical Statement

The individual featured in the wearable sensor experiment images within this manuscript is the corresponding author herself. All participants have been fully informed about the study and have provided consent for their involvement in the experimental testing. Ethical approvals were obtained from Hanyang University and Lanzhou University of Technology. Additionally, the portrait displayed in the manuscript is of the corresponding author, who has granted permission for its use. All participants have consented to the use of their images in this publication.

## 2 Results and Discussion

### 2.1 The Working Mechanism of TP‐TENG

**Figure 2:**
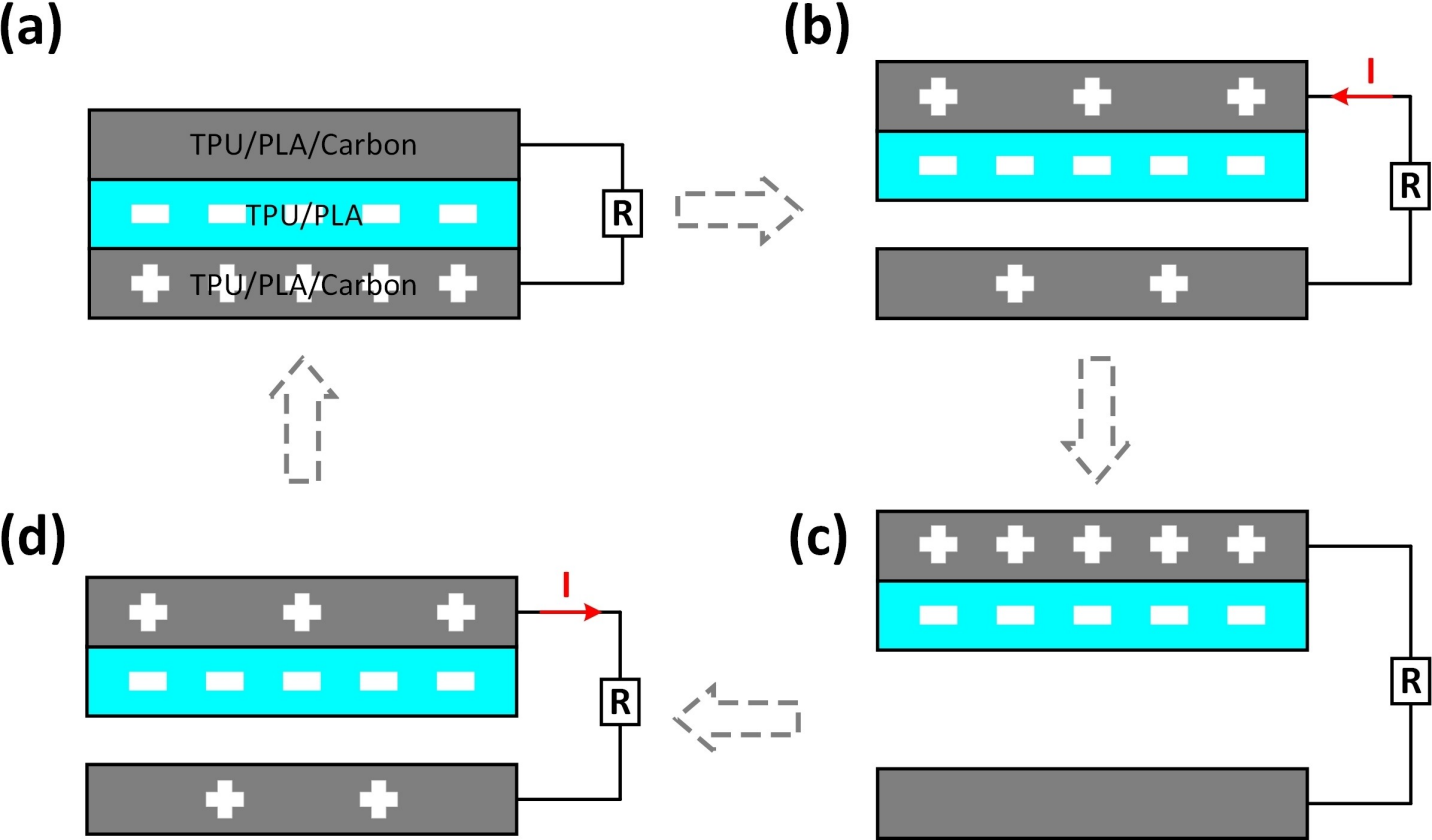
(a–d). The working mechanism of TP‐TENG.

The working mechanism of the TP‐TENG, as shown in Figure 2(a–d), mainly includes triboelectrification and electrostatic induction. The TENG will work in a contact‐separation mode when the TPU/PLA/Carbon film and TPU/PLA film with back electrode of TPU/PLA/Carbon film are contact with external circuit, and due to the introduction of carbon, TPU/PLA/Carbon film has excellent positive triboelectric properties, as present in Figure 2(a). When TPU/PLA/Carbon film and TPU/PLA film generate gaps under external forces, electrons will flow in the circuit and generate pulse currents (Figure 2(b)). When the maximum separation distance occurs between TPU/PLA/Carbon film and TPU/PLA film, TP‐TENG will no longer generate current due to the electrostatic field being in equilibrium, as illustrated in Figure 2(c). When the external force acts on TPU/PLA/Carbon film and TPU/PLA film again, directional current will be generated between the electrodes in TP‐TENG (Figure 2(d)). This contact separation reciprocating motion mechanism is very compatible with human motion, and the good compatibility between the TPU/PLA/Carbon film and TPU/PLA film film and the human body block endows the TP‐TENG device with application value in the sports monitoring field.

### 2.2 The Output Performance of TP‐TENG

**Figure 3:**
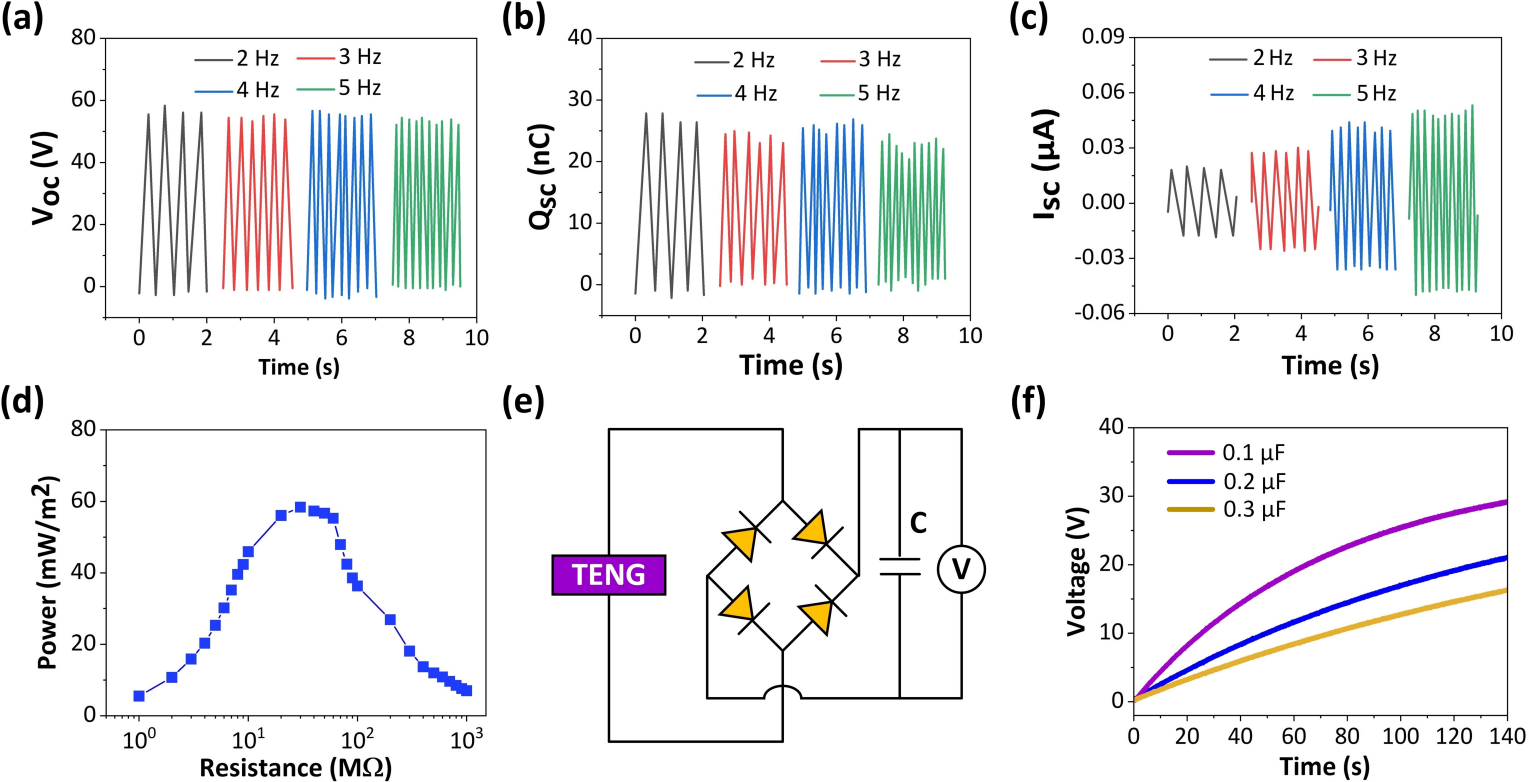
The (a) *V_oc_*, (b) *Q_sc_*, and (c) *I_sc_* of TP‐TENG under various frequencies. (d) The output power of TP‐TENG with different resistances. (e) The rectifier circuit of charging the capacitor by using TP‐TENG. (f) The charging curves of different capacitors by using TP‐TENG.

In this work, a linear motor is used to provide continuous power to TP‐TENG, enabling TP‐TENG to achieve periodic contact separation motion, thereby evaluating the output performance of TP‐TENG devices. The effective contact area of the TP‐TENG device is 2 cm×2 cm, the maximum movement displacement provided by the linear motor is 2 cm, and the contact force provided by the linear motor is 20 N. Linear motors can generate mechanical motion at different frequencies from 2 Hz to 5 Hz, used to study the impact of mechanical motion frequency on TP‐TENG output performance. The *V_OC_* and *Q_SC_* mainly depend on the triboelectric charge density, and they do not exhibit noticeable variation, as shown in Figure 3(a, b). The current means the number of charges passing through the cross section per unit time, so the *I_SC_* illustrated in Figure 3(c) increases clearly. The output under different resistances is used to evaluate the effective output performance of the TP‐TENG. As illustrated in Figure 3(d), the peak power density is 58.38 mW m^−2^ when the load resistance of 30 MΩ is matched, which is better than previous works, as shown in Table 1.

**Table 1:** The performance comparison of TP‐TENG with previous similar works.[^37^, ^38^]

TENG	Eco‐friendly TENG^[37]^	Wearable TENG^[38]^	This work
Triboelectric materials	TPU@Nylon	MWCNT@TPU	TPU/PLA@TPU/PLA/Carbon
Output power density	0.03 μW/cm^2^	0.57 μW/cm^2^	5.838 μW/cm^2^

As shown in Figure 3(e), the TP‐TENG can convert mechanical energy into electrical energy by organizing circuits and storing it in small capacitors to provide further power demand for electrical appliances. As demonstrated in Figure 3(f), the results of TP‐TENG charging capacitors with different capacitance values (from 0.1 μF to 0.3 μF) indicate that their charging rate decreases with the increase of capacitance value. The stored electric energy in capacitors can sustainably supply power for wearable electronics. According to the analyses, we can find that the designed TP‐TENG show great potential in future wearable electronics.

**Figure 4:**
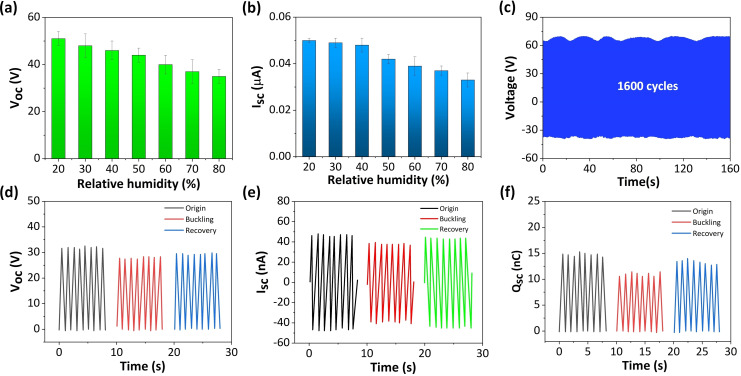
The (a) *V_oc_* and (b) *I_sc_* of TP‐TENG under various relative humidity. (c) The stability and durability testing of TP‐TENG under Nearly 1600 Cycles. The (d) *V_oc_*, (e) *I_sc_*, and (f) *Q_sc_* of TP‐TENG before and after bucking.

Moreover, electronic devices have certain requirements for humidity in the living environment, which can affect the performance of electronic devices. Hence, environmental humidity can affect the output of TENG devices. Based on this, we investigated the effect of environmental relative humidity on the output performance of TP‐TENG under the same experimental conditions except for humidity. The experimental results in Figure 4(a, b) indicate that as the relative humidity increases, the output of TP‐TENG will correspondingly decrease. This is because the increase in humidity leads to the loss of frictional charges, which in turn leads to the degradation of TP‐TENG output performance. The durability and output stability of the TENG are essential elements in practical applications. Finally, a testing platform is used to provide continuous and stable pressure signals for the prepared TP‐TENG. After approximately 1600 cycles of testing, the voltage output signals at the beginning and end of the experiment were intercepted (Figure 4(c)). It can be found that after 1600 cycles of experiments, the output voltage has remained around 65 V without a significant decrease, which further proves its good durability and development potential in practical environmental applications. The output stability of the TENG in the buckling process was also measured. Figure 4(d–f) show obvious decreases in *I_SC_*, *Q_SC_*, and *V_OC_* when the TP‐TENG devices encounter wrinkles. Specifically, the *I_SC_*, *Q_SC_*, and *V_OC_* reduce to almost 82 %, 74 %, and 87 % of their original values, respectively. Nevertheless, when TP‐TENG encounters wrinkles and continues to work for a period of time, its output performance will tend to stabilize. And the *V_OC_*, *Q_SC_*, and *I_SC_* increase to 91 %, 92 %, and 92 %, respectively, almost the same as their original values. This is because the surface of TPU/PLA/Carbon film and TPU/PLA film with good flexibility will recover to its initial state after continuous operation, demonstrating excellent usability.

### 2.3 Application in the Basketball Motions

**Figure 5:**
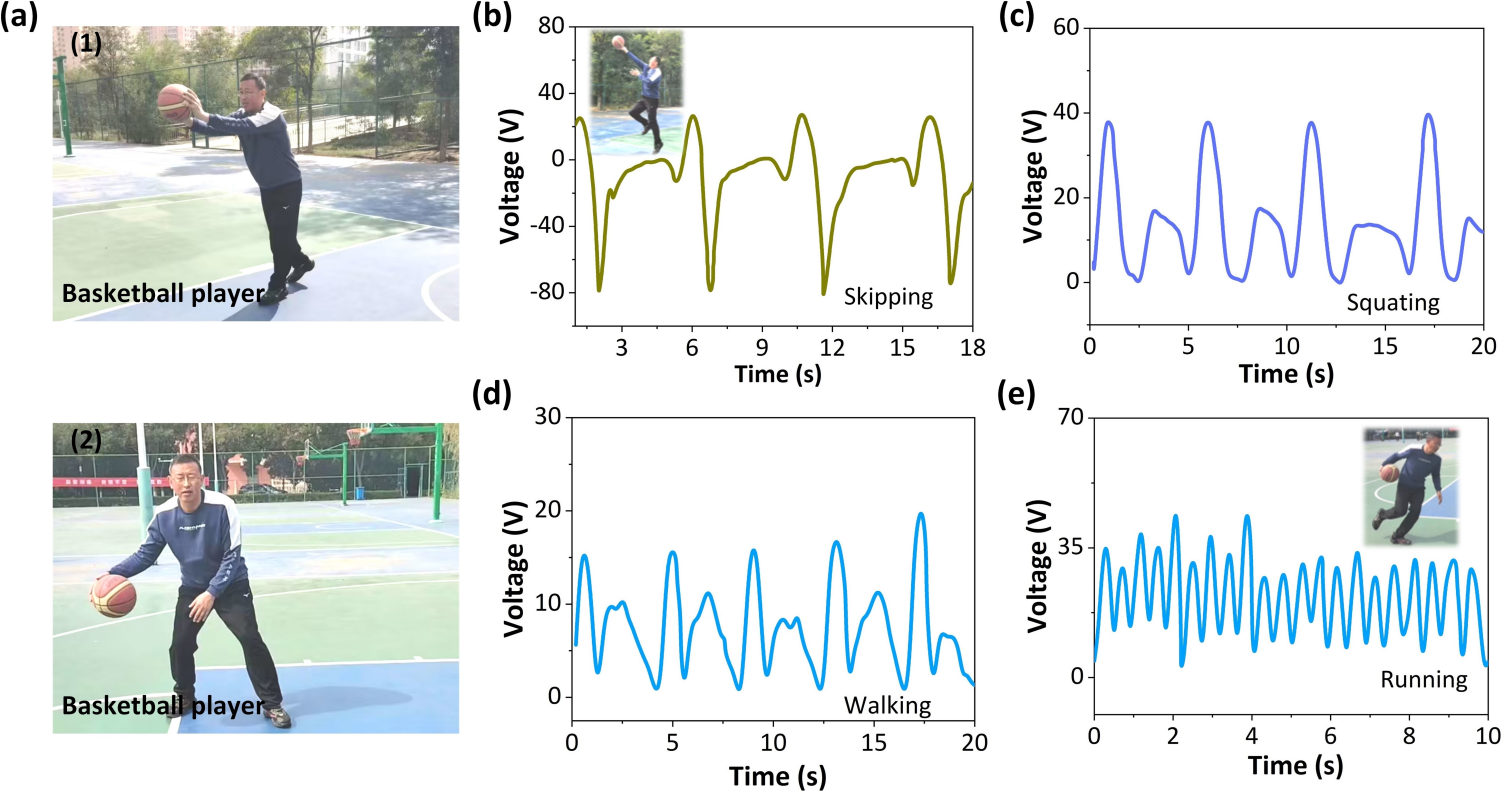
(a1, a2) The picture of basketball with different basketball posture. The output siganl of TP‐TENG sensor different basketball posture, including (b) skipping, (c) squatting, (d) walking, (e) running.

There is diversity and complexity in human movement. By integrating flexible TP‐TENG on the skin of the moving joints, human movement will drive the relative separation and contact between the frictional layers in TP‐TENG, thereby generating electrical signals that match human movement and achieving sensing of human movement. Basketball, as a popular group competitive sport, has a positive promoting effect on the comprehensive development of athletes’ physical and psychological qualities. However, the traditional basketball training mode has certain shortcomings in cultivating athletes’ basketball skills. The traditional training methods focus on imparting basketball skills, but neglect the cultivation of athletes’ awareness and awareness of the competition environment and situation. These reflections on the environment and form will be reflected in the athlete's posture and movements. Hence, monitoring the posture of basketball will help improve training methods, as demonstrated in Figure 5(a1, a2). The self‐powered TP‐TENG can monitor human movements, showing great potential in managing the security of human physical exercise. On account of such excellent sensitivity and stability, the TP‐TENG sensor can detect all types of human motions. For instance, the TP‐TENG sensor was attached to the right knee, of which the generated electrical signals could monitor different gait statements. During walking, different current signals are generated, and their wave‐forms correspond well to specific postures, including lifting the right knee, placing the right leg on the ground, extending the right knee, and moving the right knee backwards. Figure 5(b–e) shows that the TP‐TENG sensor can intelligently test and recognize human posture in different basketball motion posture, including skipping, squatting, walking, running. Clear signal differences help to identify basketball posture and provide a data basis for improving training methods in the later stages. For example, the difference between slow running and fast running can be made by the frequency of the signal generated by TP‐TENG, that is, the frequency of the sensing signal generated by TP‐TENG during slow running is lower than the frequency of the sensing signal generated by TP‐TENG during fast running.

## 3 Conclusions

In summary, a TPU/PLA 3D printing film‐based TP‐TENG was proposed to monitor basketball player posture for training effectiveness evaluation. By introducing carbon fibers, TPU/PLA/Carbon film with conductive and positive triboelectric properties was prepared to serves as both triboelectric layer and conductive electrode. It can be found that after 1600 cycles of experiments, the output voltage has remained around 65 V without a significant decrease, which further proves its good durability and development potential in practical environmental applications. According to results, the peak power density is 58.38 mW m^−2^ when the load resistance of 30 MΩ is matched. Nevertheless, when TP‐TENG encounters wrinkles and continues to work for a period of time, its output performance will tend to stabilize. And the open‐circuit voltage (*V_OC_*), transfer charge (*Q_SC_*), and short‐circuit current (*I_SC_*) increase to 91 %, 92 %, and 92 %, respectively, almost the same as their original values. Furthermore, the TP‐TENG sensor can intelligently test and recognize human posture in different basketball motion posture, including skipping, squatting, walking, running.

## Conflict of Interests

The authors declare no conflict of interest.

4

## Data Availability

The data that support the findings of this study are available on request from the corresponding author. The data are not publicly available due to privacy or ethical restrictions.
